# *IPCEF1*: Expression Patterns, Clinical Correlates and New Target of Papillary Thyroid Carcinoma

**DOI:** 10.7150/jca.98470

**Published:** 2024-10-21

**Authors:** Dechao Yin, Kun Wang, Junyu Zhao, Jinming Yao, Xiaofang Han, Bo Yan, Jianjun Dong, Lin Liao

**Affiliations:** 1 Department of Endocrinology and Metabology, Shandong Provincial Qianfoshan Hospital, Shandong University, Jinan, Shandong, China.; 2 Department of Endocrinology and Metabology, The Second People's Hospital of Hefei, Hefei Hospital Affiliated to Anhui Medical University, Hefei, Anhui, China.; 3 Department of Endocrinology and Metabology, Liaocheng People's Hospital, Liaocheng, Shandong, China.; 4 Department of Endocrinology and Metabology, The First Affiliated Hospital of Shandong First Medical University & Shandong Provincial Qianfoshan Hospital, Jinan, Shandong, China.; 5 Department of Endocrinology, Qilu Hospital of Shandong University, Jinan, Shandong, China.

**Keywords:** papillary thyroid carcinoma, *IPCEF1*, JAK/STAT signaling pathway, immune infiltration, prognosis, therapeutic targets

## Abstract

**Introduction:** Despite the generally favorable prognosis of PTC (Papillary Thyroid Carcinoma), it can still exhibit aggressive behavior and lead to patient mortality. *IPCEF1* (interaction protein for cytohesin exchange factors 1) has emerged as a critical player in cell signaling related to proliferation and migration in cancer progression.

**Objective:** Our research aimed to determine whether *IPCEF1* is a key gene in PTC, elucidate its possible molecular mechanisms and ultimately search for new targets.

**Methods:** This research utilized four gene expression array datasets and TCGA database to examine the role of *IPCEF1* in PTC. Differential gene expression analysis, survival analysis, KEGG and GO enrichment and immune cell infiltration correlations were realized by bioinformatic methods. The expressions of *IPCEF1* in PTC tissues were examined by IHC and the proliferation, migration, cell cycles of PTC cells were examined by CCK8, transwell and flow cytometry.

**Results:** *IPCEF1* had lower expression in PTC tumor tissues and its lower expression might lead to worse T/N stage and DFS/ PFS, which is perhaps related to its regulation of the JAK/STAT signaling pathway and immune microenvironment (macrophage and Tregs). *IPCEF1* reduced the proliferation and migration ability of PTC cells, which is consistent with our clinical observations. Besides, we also found that high expression level of *IPCEF1* lead to cell cycle arrest in the S or G2 phase, which ultimately reduced cell growth and proliferation.

**Conclusion:** *IPCEF1* is a cancer suppressor gene in the progression of PTC, influencing patient survival and prognosis through modulation of immune infiltration and signaling pathways.

## Introduction

Papillary Thyroid Cancer (PTC), accounting for approximately 90% of all thyroid cancers, is a prevalent endocrine malignancy with an incidence that has been increasing in recent years 1. The increase in thyroid cancer incidence was almost entirely due to the increase of PTC. The reasons behind the increase are complex, multifactorial, and incompletely understood. Despite the generally indolent nature of PTC and its relatively low mortality rate, the 10-year rates for local recurrence and distant metastasis remain concerning, at approximately 20% and 10%, respectively, significantly affecting patient survival 2,3. In the realm of advanced thyroid cancers, including recurrent or metastatic cases, the landscape of targeted therapies has evolved rapidly, and China has seen the approval of various targeted drugs with distinct mechanisms of action for these malignancies 4. However, the severe adverse effects associated with such therapies have curtailed their broader application, necessitating the pursuit of new therapeutic targets and agents.

Emerging evidence has spotlighted the critical role of *IPCEF1* (interaction protein for cytohesin exchange factors 1) in various physiological and pathological processes. This protein is recognized for its intricate involvement in cell signaling pathways, particularly those related to cell proliferation and migration 5. Recent studies have suggested a possible link between *IPCEF1* and cancer progression, although the exact mechanisms and implications remain largely unexplored 6-8. Notably, the involvement of *IPCEF1* in thyroid papillary carcinoma remains an uncharted territory deserving of comprehensive investigation.

Our research aimed to determine whether *IPCEF1* is a key gene in PTC through the analysis of multiple gene expression array datasets, verification of its expression in clinical tissues, exploring the impact of its expression level on the proliferation, migration, cell cycle of PTC cells, elucidating its possible molecular mechanisms and ultimately searching for new targets.

## Materials and methods

### Data collection and microarray data

The GEO database (https://www.ncbi.nlm.nih.gov/geo/) 9 was utilized to access public studies available up to September 15, 2023. The search employed the following keywords: “papillary thyroid cancer,” “Homo sapiens” (as the organism), and “array expression profile” (as the research type). The inclusion criteria were: (1) patients diagnosed with PTC; (2) gene expression profiling of mRNA; and (3) availability of sufficient information for analysis. Following a systematic review, four gene expression profiles (GSE29265, GSE60542, GSE151179, and GSE33630) were selected for analysis. The platforms used for gene profiling of these datasets were GPL570 (for GSE29265, GSE60542, and GSE33630) and GPL23159 (for GSE151179), all of which are Affymetrix microarrays.

### Data analyses of differentially expressed genes (DEGs)

Microarray data were acquired from the GEO database. The raw data were downloaded in MINiML file format. Differential expression analysis of mRNA was conducted using the limma package within R software 10. The adjusted P-value was calculated to mitigate false positives in the GEO datasets, with thresholds set at “Adjusted P<0.05” and a log-fold change greater than 1 or less than -1 to define significant differential expression. The Sangerbox tool (http://sangerbox.com/) 10 facilitated the identification of common DEGs across the four datasets and the creation of Venn diagrams.

### Expression of DEGs in THCA

Following the identification of DEGs in the GEO datasets, we analyzed the expression levels of these genes in thyroid cancer within the TCGA database. This included an examination of DEG expression across various tumor grades (T grades), nodal involvement (N grades), and TNM stages. RNA-sequencing data (level 3) and corresponding clinical information for thyroid carcinoma (THCA) were sourced from the TCGA database (https://portal.gdc.cancer.gov). Concurrently, the latest GTEx datasets (V8) were retrieved from the GTEx portal (https://www.gtexportal.org/home/datasets). All statistical analyses were performed using R software version 4.0.3 (R Foundation for Statistical Computing, Vienna, Austria), considering a P-value <0.05 as indicative of statistical significance. Data visualization, including the generation of Venn diagrams, was achieved using the “ggplot2” R package and the Sangerbox software 11.

### Survival analysis of DEGs in THCA

Kaplan-Meier (KM) survival analysis and the log-rank test were employed to compare the survival differences between two groups, considering overall survival (OS), disease-free survival (DFS), and progression-free survival (PFS). For the Kaplan-Meier curves, p-values and hazard ratios (HR) with 95% confidence intervals (CI) were calculated using log-rank tests and univariate Cox proportional hazards regression models. All analytical methods and R package implementations were conducted using R software version 4.0.3 (The R Foundation for Statistical Computing, 2020). A p-value of less than 0.05 was considered statistically significant.

### Immunohistochemical analysis (IHC)

The expression of *IPCEF1* was examined in tumor tissues (20 samples) and adjacent normal tissues (20 samples) from 20 PTC patients, acquired from The Second People's Hospital of Hefei. Tissue sections (4 μm thick) from formalin-fixed, paraffin-embedded (FFPE) samples were deparaffinized in xylene and rehydrated through an ethanol-water gradient. Antigen retrieval was performed under high temperature and pressure for 15 minutes using an antigen repair solution. Sections were then incubated with 3% hydrogen peroxide for 10 minutes to quench endogenous peroxidase activity. Overnight incubation with *IPCEF1* antibody (1:50, ab272599, Abcam, Cambridge, USA) was carried out at 4°C. Following PBS washes, slides were incubated with goat anti-rabbit secondary antibody conjugated to peroxidase (1:100, ab205718, Abcam, Cambridge, USA) at 37°C for 30 minutes. Peroxidase activity was visualized using DAB Peroxidase Substrate (DA1010, Solarbio, Beijing), and slides were subsequently counterstained with hematoxylin. The protocol was approved by the Research Ethics Committee of The Second People's Hospital of Hefei, Anhui, China, and informed consent was obtained from all patients.

### Cell culture

BHP10-3 and TPC-1 were given away from Endocrinology Laboratory of Shandong Province Qianfoshan Hospital. Cells were cultured in DMEM medium (Gibco, 11995065) supplemented with 10% fetal bovine serum (Abwbio, AB-FBS0500) in 5% CO2 and 37°C.

### Reagents

The following antibodies were used, E-cadherin (20874-1-AP), N-cadherin (22018-1-AP), MMP9 (10375-2-AP), SNAL1(13099-1-AP), P21 (10355-1-AP), GAPDH (60004-1-Ig) were purchased from Proteintech (Wuhan, China). P27 (ET1608-61), cdc2 (ET1605-54), Cyclin B1 (ET1608-27), Cyclin D1 (ET1601-31), were purchased from huabio (Hangzhou, China). *IPCEF1* (ab272599), Cdc25C (ab32444) and the secondary antibodies (ab205718, ab205719) were procured from Abcam (Cambridge, UK).

### Lentiviral transfection

Cells were seeded in a 6-well plate at a density of 5×10^4^ cells per well and incubated for 24 hours. When the cell confluence reached 30%, TPC-1/BHP10-3 cells were separately infected with overexpressed/RNA interference lentiviruses targeting *IPCEF1*. After 16 hours of infection, the medium was replaced with complete culture medium and cells were further cultured for 72 hours. Infection efficiency was assessed, and cells were selected with 2 μg/mL puromycin (Abcam, ab141453) to ensure the establishment of stable *IPCEF1*-interfered cell lines.

### CCK-8 assay

Cells were seeded in a 96-well plate at a density of 3000 cells per well, with medium changed every 2 days. At fixed time intervals, 10% CCK-8 reagent (Beyotime, C0039) was added and incubated at 37°C for 2 hours. The OD value was measured at a wavelength of 450 nanometers using a microplate reader. Cell growth was monitored continuously for 7 days to analyze cell proliferation.

### Colony formation

Cells were seeded in a 6-well plate at a density of 600 cells per well, with medium changed every 3 days. After 2 weeks, cells were fixed with 4% paraformaldehyde for 30 minutes, stained with 0.1% crystal violet (Solarbio, G1063) for 15 minutes, and the survival rate of clones was calculated.

### Cell migration assay

Cells in the logarithmic growth phase were digested and resuspended in serum-free medium to a final volume. The cell density was adjusted to 5 × 10^5^ cells/mL. 200 μL of cell suspension was added to the upper chamber of a Transwell, while medium containing 10% FBS was added to the lower chamber of a 24-well plate, and co-cultured for 24 hours. Subsequently, the cells were fixed with a 4% paraformaldehyde solution for 30 minutes, the upper chamber cells were removed, and the migratory cells on the bottom of the Transwell were stained with crystal violet. Five fields of view were examined under a microscope after staining to analyze the migration rate.

### Western blot analysis

Cellular proteins were lysed and collected using RIPA lysis buffer containing a protease inhibitor (Beyotime, P1045). The protein concentration was determined using a BCA protein quantification kit (Beyotime, P0010). Following separation by SDS-PAGE (10%), the protein samples were transferred onto a PVDF membrane (Millipore, ISEQ00010). The membrane was then incubated in 5% blocking solution for 1 hour, followed by overnight incubation at 4°C with the primary antibody of interest, and subsequent 1h incubation at room temperature with the secondary antibody. Protein bands were visualized and analyzed using the ECL chemiluminescence method (Beyotime, P0018M).

### Cell cycle analysis

After incubating the cells in a 6-well plate for 24 hours, they were harvested using trypsin without EDTA, washed with PBS, and centrifuged at 1000 rpm for 5 minutes. The cells were then fixed in 70% ice-cold ethanol for 24 hours, washed with PBS to remove the fixative, and resuspended in 100 μl RNase A before being incubated in a 37°C water bath for 30 minutes. Subsequently, 400 µl of PI staining solution was added, mixed thoroughly, and incubated in the dark at 4°C for 30 minutes. The cell cycle was then analyzed using flow cytometry.

### Correlation analysis and KEGG pathway, GO enrichment of top 50 genes

Correlation between two genes was assessed using the ggstatsplot package in R software. Spearman's correlation analysis was utilized to evaluate the association between quantitative variables that do not follow a normal distribution. P-values less than 0.05 were deemed statistically significant (*P < 0.05). The top 50 genes were selected for further Kyoto Encyclopedia of Genes and Genomes (KEGG) and Gene Ontology (GO) analyses.

KEGG (www.kegg.jp/kegg/kegg1.html) 12 and DAVID 13 databases were utilized for conducting KEGG pathway and Gene Ontology (GO) enrichment analyses. GO analysis encompassed three domains: biological process, cellular component, and molecular function. The criteria for selection included a minimum of 2 differentially expressed genes (DEGs) per term, with a threshold for statistical significance set at P < 0.05.

### Immune analysis

For robust immune score evaluation, we applied the “immuneeconv” R package, which amalgamates six of the most recent algorithms-TIMER, xCell, MCP-counter, CIBERSORT, EPIC, and QuanTIseq. Each of these algorithms has been benchmarked and offers unique advantages. In this study, the CIBERSORT algorithm was specifically used to calculate the immune cell scores. The “ggstatsplot” package in R software facilitated the creation of correlation plots between gene expression and immune scores. Spearman's correlation analysis was employed to describe the associations between quantitative variables that deviated from normal distribution. P-values less than 0.05 were considered statistically significant (*P<0.05). All analyses and visualization, including the use of the “ggplot2” and “heatmap” packages, were performed with R version 4.0.3, provided by the R Foundation for Statistical Computing (2020).

### Statistical analysis

For categorical data, the chi-square test was used to compare proportions. The comparison of means for normally distributed data was conducted using an independent-samples t-test for two-group comparisons or one-way ANOVA for multi-group comparisons. Non-parametric tests were employed for data that did not conform to normal distribution. All statistical analyses were conducted using SPSS software version 26.0, and GraphPad Prism version 8.0 was utilized for graphical representations.

## Results

### Significantly DEGs between tumor and adjacent normal tissues of PTC in GEO database

**Figure 1:**
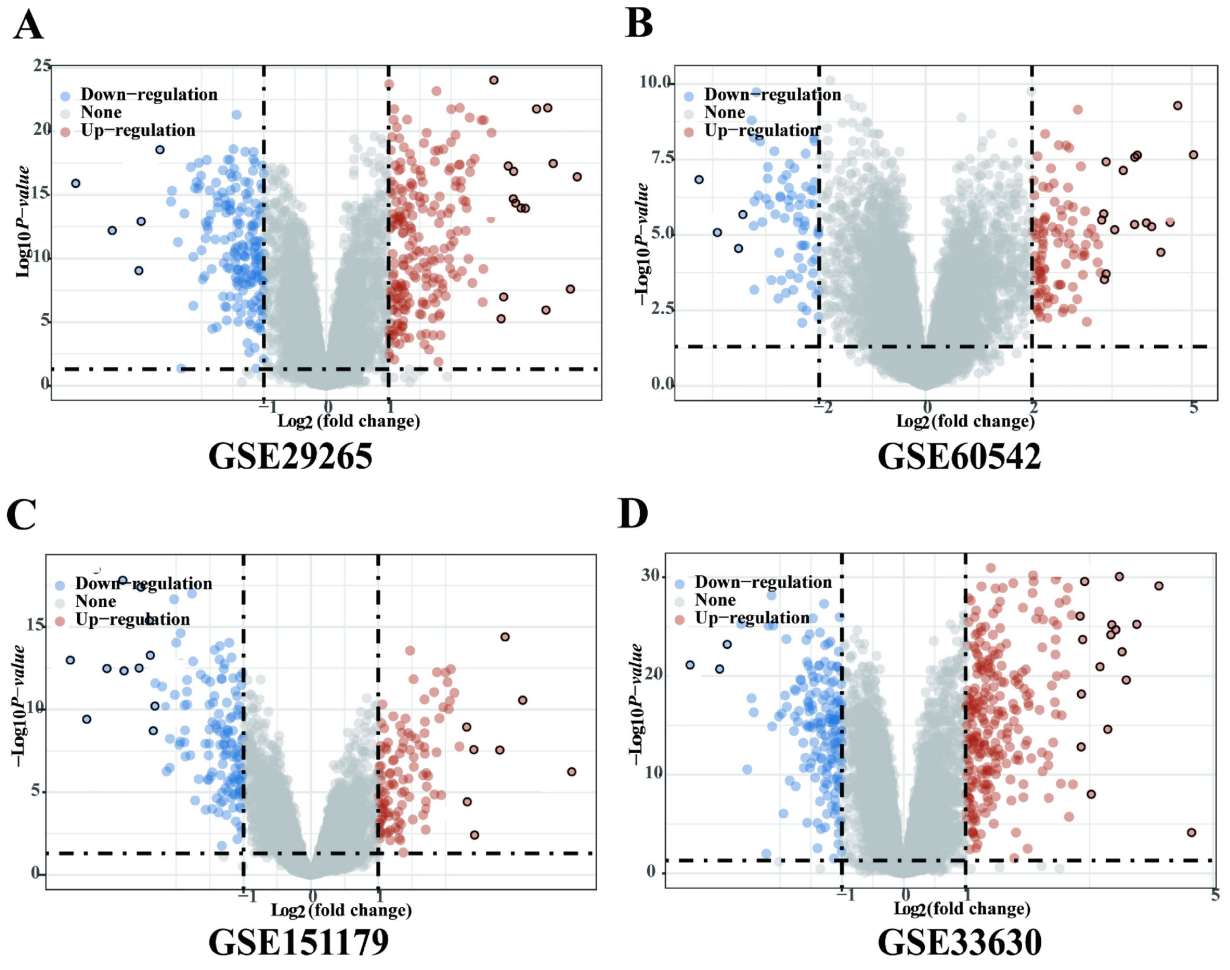
** Significantly DEGs between tumor and adjacent normal tissues of PTC in GEO database.** (A) Volcano plot for DEGs of GSE29265. (B) Volcano plot for DEGs of GSE60542. (C) Volcano plot for DEGs of GSE151179. (D) Volcano plot for DEGs of GSE33630.The x-axis shows the fold-change in gene expression between different samples, and the y-axis shows statistical significance of the differences. Significantly Up- and Down-regulated genes are filtered (|log2 (Fold Change)| > 1, P < 0.05) and highlighted in red and blue dots, respectively.

Four gene expression profiles (GSE29265, GSE60542, GSE151179 and GSE33630) were identified from the GEO database. The GSE29265 dataset contained 20 PTC tissues samples and 10 normal thyroid samples; the GSE60542 dataset included 33 PTC tissues samples and 30 normal thyroid samples; the GSE151179 dataset included 39 PTC tissues samples and 13 normal thyroid samples; the GSE33630 dataset included 49 PTC tissues samples and 43 normal thyroid samples. There were 250 upregulated and 206 downregulated DEGs in GSE29265, 225 upregulated and 188 downregulated DEGs in GSE60542, 117 upregulated and 146 downregulated DEGs in GSE151179, 270 upregulated and 170 downregulated DEGs in GSE33630 (Figure 1A-D).

### The expression of 12 intersection genes of PTC in GSE29265, GSE60542, GSE151179 and GSE33630

**Figure 2:**
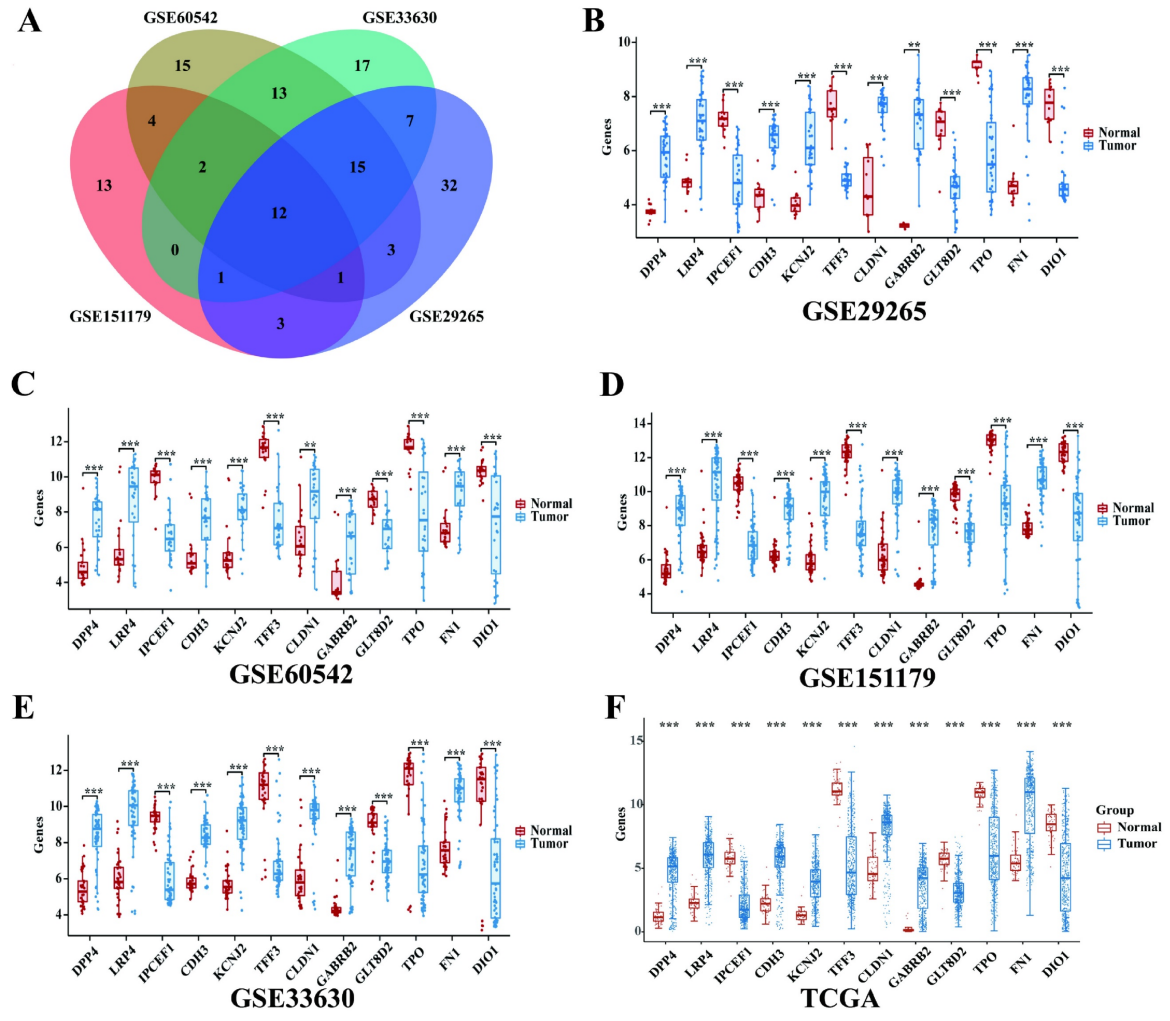
** The expression of 12 intersection genes of PTC in GSE29265, GSE60542, GSE151179, GSE33630 and TCGA database.** (A) Venn diagrams of common DGEs combined with four datasets (GSE29265, GSE60542, GSE151179 and GSE33630). (B) The expression of 12 intersection genes of PTC in GSE29265. (C) The expression of 12 intersection genes of PTC in GSE60542. (D) The expression of 12 intersection genes of PTC in GSE151179. (E) The expression of 12 intersection genes of PTC in GSE33630. (F) The expression of 12 intersection genes of PTC in TCGA database (*P < 0.05; **P < 0.01; and ***P < 0.001).

Then we found 12 significantly differentially expressed genes between PTC tissues samples and normal samples (Figure 2A) by overlapping the DEGs of the 4 GEO datasets. The 12 intersection genes were DPP4, LRP4, *IPCEF1*, CDH3, KCNJ2, TFF3, CLDN1, GABRB2, GLT8D2, TPO, FN1 and DIO1.Next, we analyzed the trend of discrepancies of the 12 genes in GSE29265, GSE60542, GSE151179 and GSE33630. The results showed that the expression of *IPCEF1*, TFF3, GLT8D2, TPO and DIO1 were downregulated in the tumor group and DPP4, LRP4, CDH3, KCNJ2, CLDN1, GABRB2, FN1 were upregulated in the tumor group (Figure 2B-E).

The mRNA expressions of 12 intersection genes were also analyzed in 397 cases of PTC tumor samples and 59 cases of normal thyroid samples. The results showed that all of the 12 intersection genes were significantly differentially expressed between PTC and normal samples and the trend of discrepancies was consistent with that in the GEO database (Figure 2F).

### DPP4, *IPCEF1*, TFF3, GABRB2, TPO, FN1 and DIO1 might play critical oncogenic roles in PTC progression

**Figure 3:**
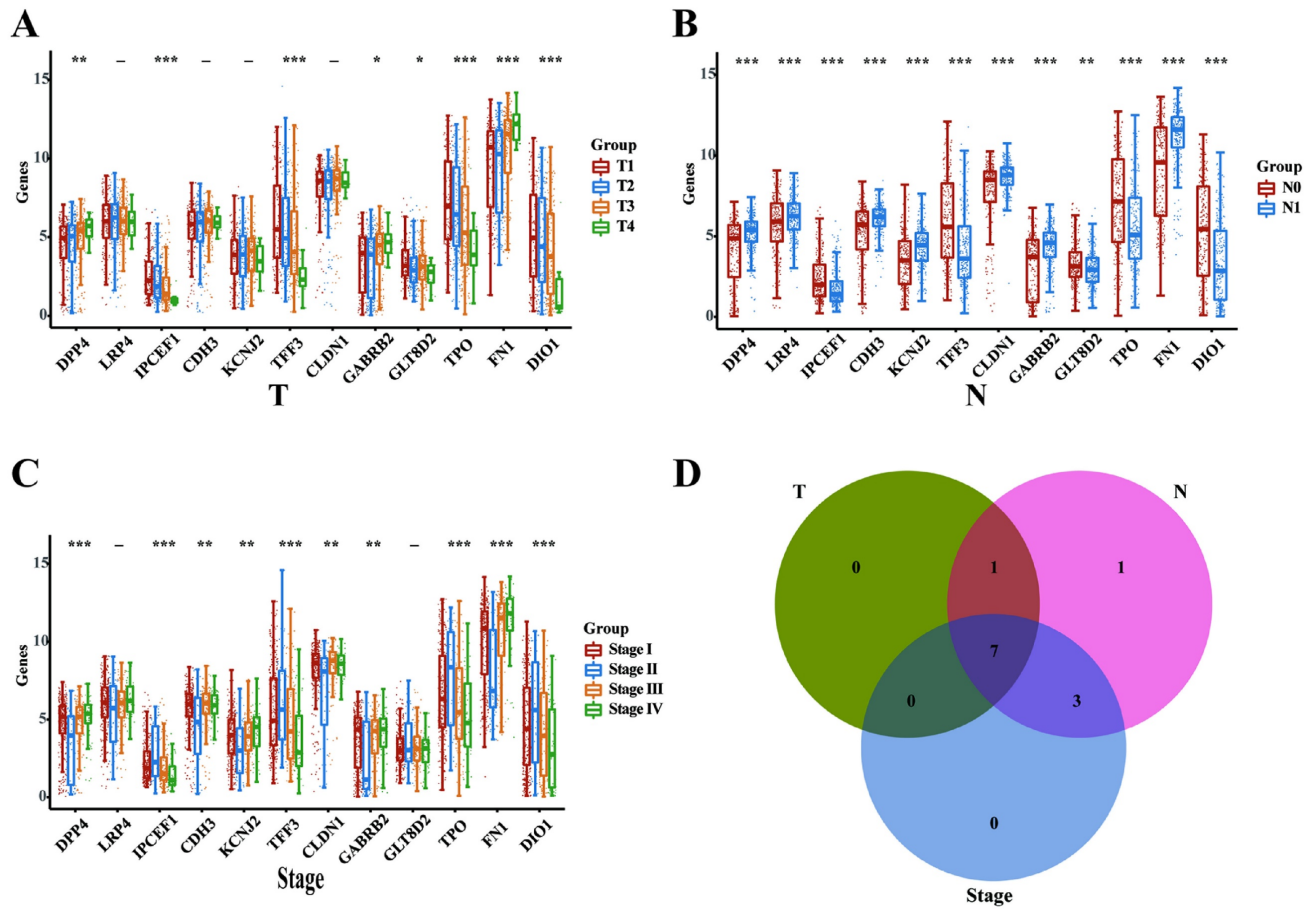
** DPP4, *IPCEF1*, TFF3, GABRB2, TPO, FN1 and DIO1 might play critical oncogenic roles in PTC progression (TCGA).** (A) The mRNA expressions of the 12 intersection genes in various T stages (8 DEGs). (B) The mRNA expressions of the 12 intersection genes in various N stages (12 DEGs). (C) The mRNA expressions of the 12 intersection genes in various TNM stages (10 DEGs). (D) The intersect of the three sets of DEGs (7 DEGs). (*P < 0.05; **P < 0.01; and ***P < 0.001).

Subsequently, we analyzed the mRNA expression levels of the 12 intersecting genes across various clinicopathological stages in the THCA database, encompassing T, N, and TNM categories. In the T categories (T1, T2, T3, and T4), we identified 8 DEGs (DPP4, *IPCEF1*, TFF3, GABRB2, GLT8D2, TPO, FN1, and DIO1; Figure 3A). In the N categories (N0 and N1), we delineated 12 DEGs (DPP4, LRP4, *IPCEF1*, CDH3, KCNJ2, TFF3, CLDN1, TPO, FN1, DIO1; Figure 3B). In the TNM categories, 10 DEGs were identified (DPP4, LRP4, *IPCEF1*, CDH3, KCNJ2, TFF3, CLDN1, TPO, FN1, and DIO1; Figure 3C). Finally, the intersection of the three sets of DEGs (Figure 3D) revealed that 7 genes (DPP4, *IPCEF1*, TFF3, GABRB2, TPO, FN1, and DIO1) may play critical oncogenic roles in PTC progression.

### Low expression of *IPCEF1* might lead to poor prognosis in PTC patients

**Figure 4:**
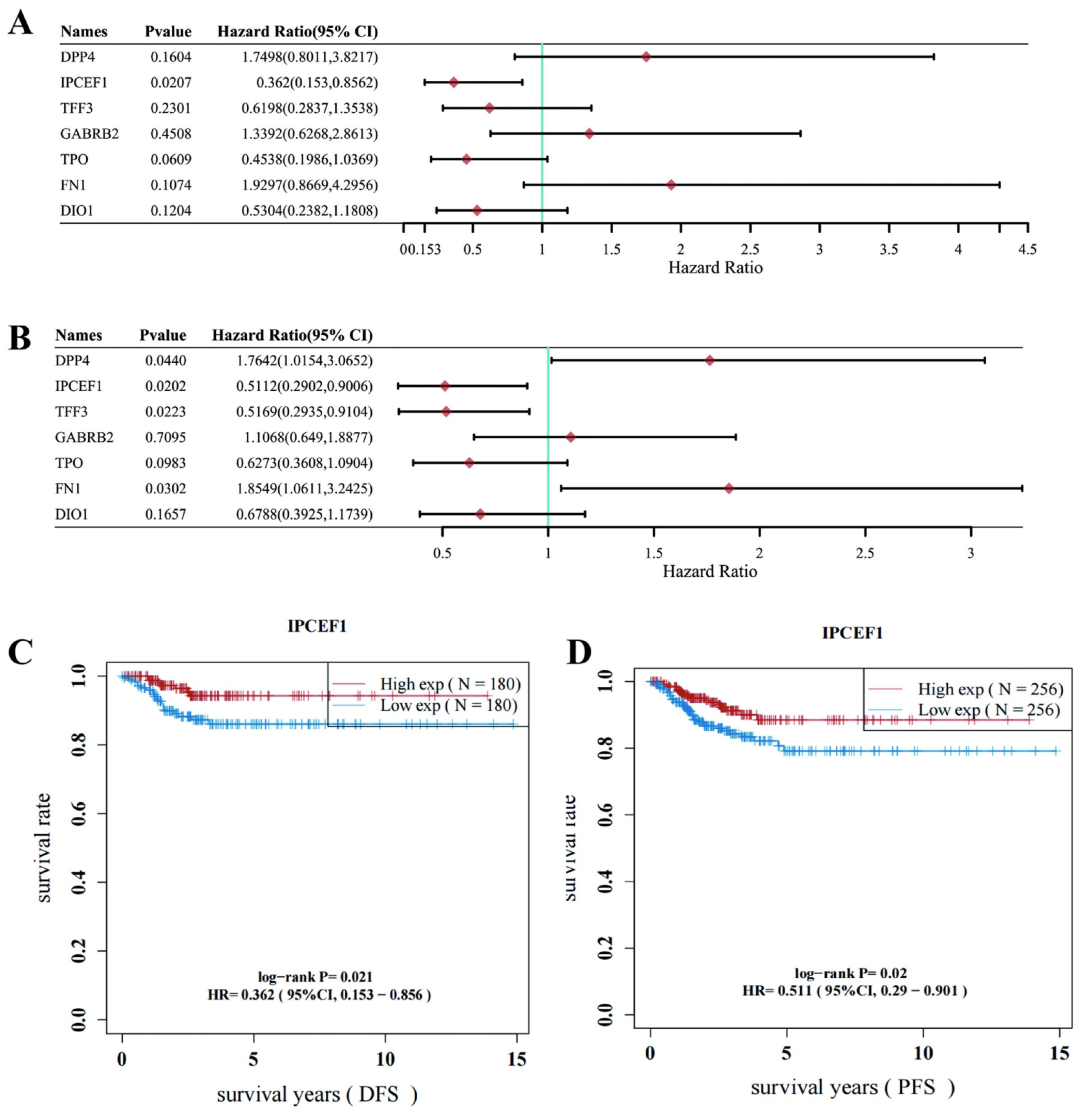
** Low expression of *IPCEF1* might lead to poor prognosis in PTC patients (TCGA).** (A) Forest plot of Disease-Free Survival (DFS) analysis of 7 DEGs for PTC patients. (B) Forest plot of Progression-Free Survival (PFS) analysis of 7 DEGs for PTC patients. (C) Disease-Free Survival (DFS) analysis curve of *IPCEF1* in PTC. (P < 0.05) (D) Progression-Free Survival (PFS) analysis curve of *IPCEF1* in PTC (P < 0.05).

The Disease-Free Survival (DFS) and Progression-Free Survival (PFS) of the 7 DEGs associated with PTC were further analyzed. Survival analysis revealed that *IPCEF1* was the sole gene displaying a significant difference in DFS for PTC, while two genes (*IPCEF1* and TFF3) showed significant differences in PFS for PTC within the THCA database (Figures 4A-B). Consequently, *IPCEF1*, which exhibited differences in both DFS and PFS, was chosen as the target gene for our study. Additionally, we presented the results of the survival analysis (DFS and PFS) for *IPCEF1* in Figures 4C and 4D, indicating that low expression of *IPCEF1* may be associated with a poor prognosis in PTC patients.

### *IPCEF1* expression was lower in PTC and was related to clinicopathological features

**Figure 5:**
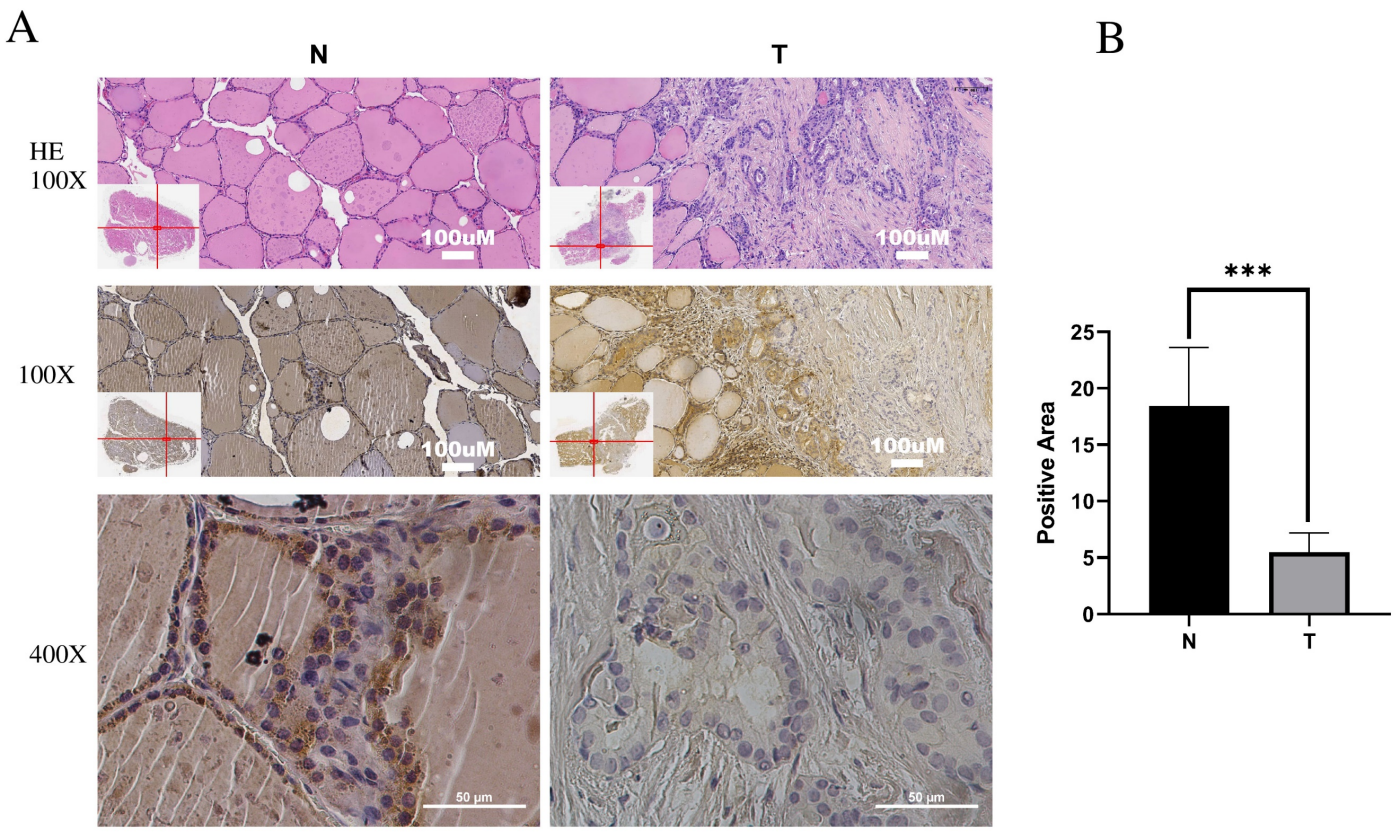
** Low expression of *IPCEF1* in PTC. (A) *IPCEF1* expression in tumor and adjacent normal tissues of PTC by immunohistochemistry.** HE, (the first line), Magnification, x100 (the second line) and x400 (the third line). (B) Quantification of positive-staining for *IPCEF1* in thyroid cancer tissues of tumor and adjacent normal tissues of PTC (***P < 0.001).

Immunohistochemistry (IHC) was employed to investigate *IPCEF1* expression in PTC tissues. The results indicated that *IPCEF1* localization was primarily observed in the cell membrane and cytoplasm (Figure 5A). Subsequently, quantification of IHC-positive areas was conducted using ImageJ analysis software and IHC_Profiler. IHC analysis revealed that the positive staining area in PTC sections (5.48%) was significantly lower than in normal tissue (18.44%; Figure 5B, P <0.01), suggesting a reduced expression of *IPCEF1* in PTC tissues. This observation supports the hypothesis that reduced *IPCEF1* expression could contribute to PTC tumorigenesis.

We then analyzed the association between *IPCEF1* expression levels and clinicopathological characteristics of PTC from TCGA database (Table 1). Patients with PTC were categorized based on low and high *IPCEF1* expression levels to assess their correlation with clinicopathological parameters, such as age, gender, N stage, T stage, M stage, and overall TNM stage. The analysis showed no significant differences in age and gender distributions between the low and high *IPCEF1* expression groups. Notably, in the low *IPCEF1* expression group, a significantly greater percentage of patients had advanced T stage (III-IV), lymph node metastases (N1), and higher TNM stages (III-IV) compared to early T stage (I-II), no lymph node metastases (N0), and lower TNM stages (I-II), respectively. The observed correlations between *IPCEF1* expression and the clinicopathological characteristics of PTC suggest that lower *IPCEF1* levels may be indicative of a higher PTC grade and the presence of lymph node metastases.

### The expression level of *IPCEF1* could influence the proliferative capacity of PTC cells

**Figure 6:**
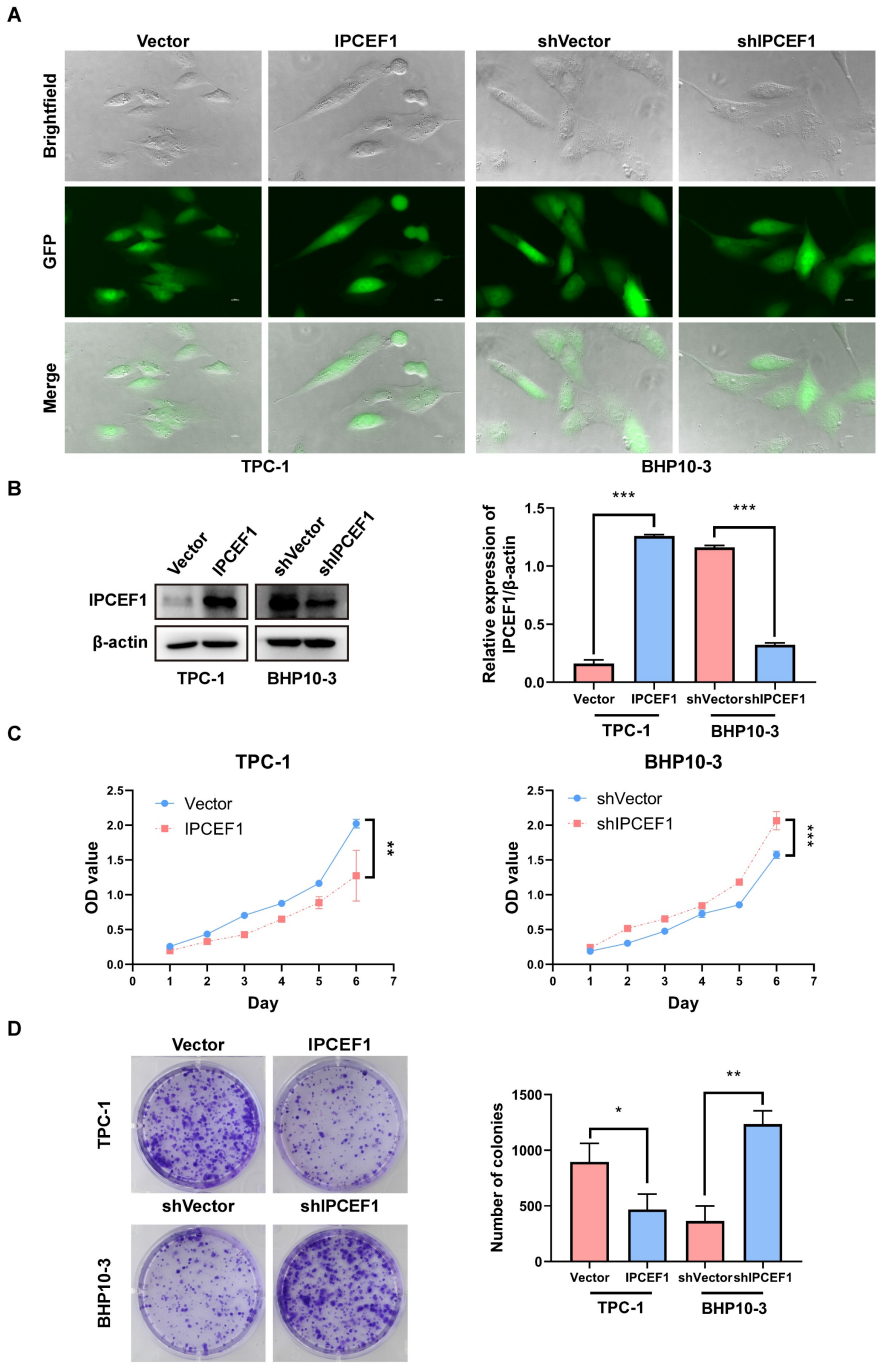
** The expression level of *IPCEF1* influences the proliferative capacity of PTC cells.** (A) Transfection of cells observed through fluorescence. (B) Transfection efficiency of cells detected by Western Blot. (C) Impact of *IPCEF1* gene alterations on the proliferation of thyroid cancer cells assessed using the CCK8 assay. (D) Influence of *IPCEF1* gene alterations on cell colony formation examined through crystal violet staining. (*P < 0.05, **P < 0.01, ***P < 0.001).

Preliminary observation of GFP fluorescence confirmed successful transfection of *IPCEF1* in PTC cell lines (Figure 6A). Western blot analysis revealed elevated levels of *IPCEF1* expression in the *IPCEF1* group (which was infected with overexpressed lentiviruses targeting *IPCEF1*) compared to Vector cells, while the sh*IPCEF1* group (which was infected with RNA interference lentiviruses targeting *IPCEF1*) displayed the opposite trend (Figure 6B). CCK8 results showed that overexpression of *IPCEF1* reduced the proliferation of PTC cells, while silencing low expression of *IPCEF1* significantly promoted the proliferation of PTC cells (Figure 6C). Colony formation experiments compared the clonogenic capacity of cells in each group (Figure 6D), where the *IPCEF1* group exhibited significantly fewer colony numbers than the Vector group, and the sh*IPCEF1* group had more colonies, consistent with the growth curve data mentioned earlier.

### The expression level of *IPCEF1* could influence the migration ability of PTC cells

**Figure 7:**
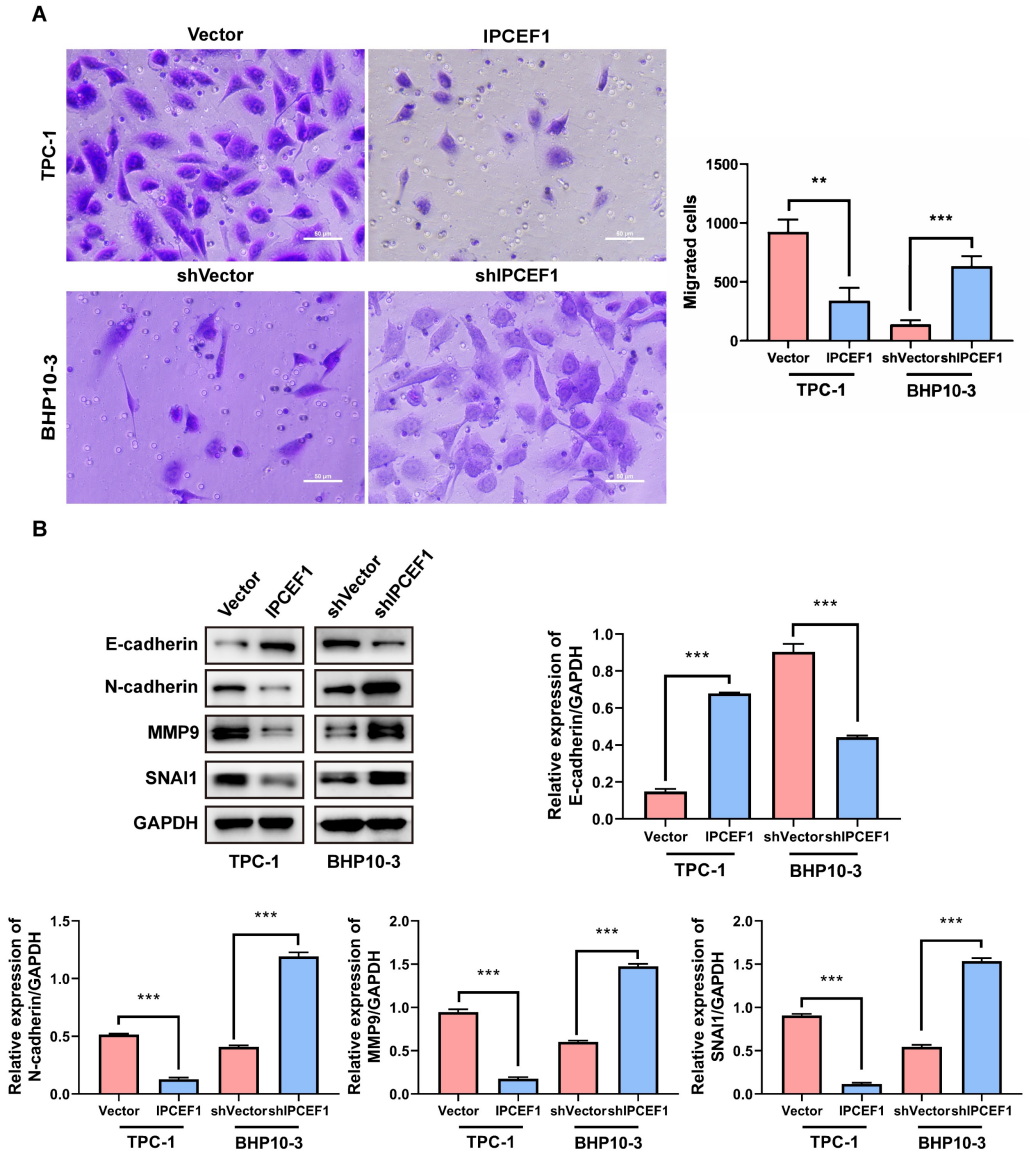
** The expression level of *IPCEF1* influences the migration ability of PTC cells.** (A) Transwell assay to detect changes in cell migration ability and perform analysis. (B) Western blot analysis of relevant indicators of cell migration and quantitative analysis. (**P < 0.01, ***P < 0.001).

The transwell experiment was employed to examine the changes in cell migration ability following alterations in *IPCEF1* expression levels. The results revealed that compared to the control group, cells overexpressing *IPCEF1* exhibited weakened migration ability, while cells with silenced low expression of *IPCEF1* showed significantly enhanced migration ability (Figure 7A). Furthermore, Western Blot analysis was conducted to assess the impact of changes in *IPCEF1* expression levels on the expression levels of EMT epithelial marker E-cadherin, mesenchymal marker N-cadherin, and migration-related indicators MMP9 and SNAI1 in each group. The results indicated that, compared to the Vector group, the *IPCEF1* group upregulated E-cadherin and downregulated N-cadherin, MMP9, and SNAI1 expression levels. Conversely, the sh*IPCEF1* group exhibited the opposite pattern (Figure 7B). This suggested a correlation between changes in *IPCEF1* expression levels and EMT.

### The expression level of *IPCEF1* could influence the cell cycle of PTC cells

**Figure 8:**
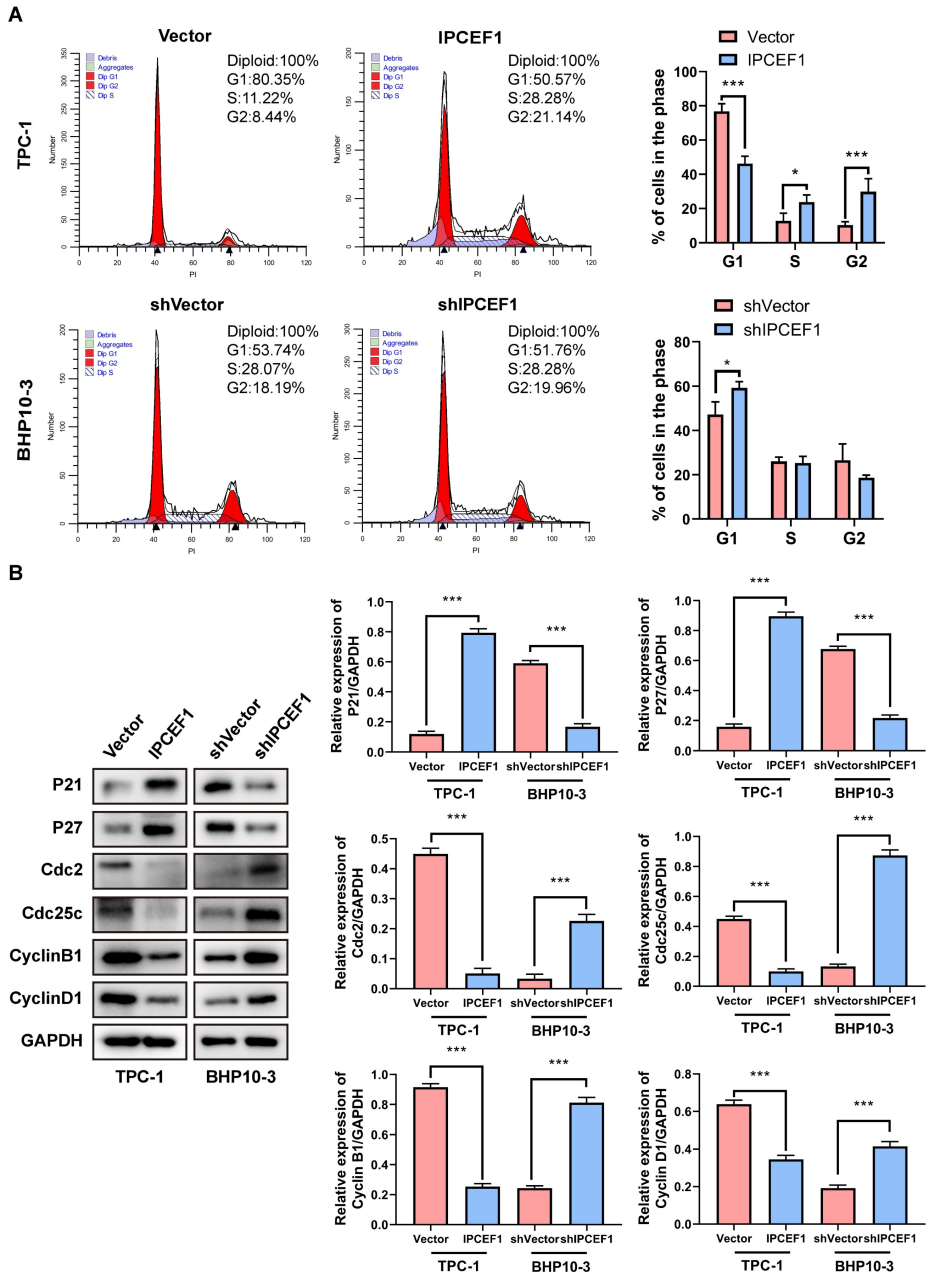
** The expression level of *IPCEF1* can influence the cell cycle of PTC cells.** (A) Flow cytometry to detect changes in the cell cycle of PTC cells and conduct analysis. (B) Western blot to examine the expression levels of cell cycle-related indicators and perform quantitative analysis (*P < 0.05, **P < 0.01, ***P < 0.001).

Flow cytometry was utilized to examine the impact of changes in *IPCEF1* expression levels on the cell cycle of different cell groups. The results indicated that compared to the Vector group, cells overexpressing *IPCEF1* showed a decrease in the proportion of G1 phase cells, with a significant increase in the proportions of S and G2 phase cells. In comparison to the shVector group, the sh*IPCEF1* group exhibited an increase in the proportion of G1 phase cells, with no significant differences in other phase proportions (Figure 8A). Furthermore, Western blot analysis was conducted to determine whether the expression levels of cell cycle-related indicators P21, P27, cdc2, Cdc25C, CyclinB1, and CyclinD1 were altered in each group of cells. The results revealed that, compared to the Vector group, the *IPCEF1* group upregulated P21 and P27, while downregulating the expression levels of cdc2, Cdc25C, CyclinB1, and CyclinD1. Conversely, the sh*IPCEF1* group showed opposite results (Figure 8B). This suggested that changes in *IPCEF1* expression levels could influence the cell cycle of PTC cells.

### Correlation analyses, KEGG pathway and GO enrichment analyses

**Figure 9:**
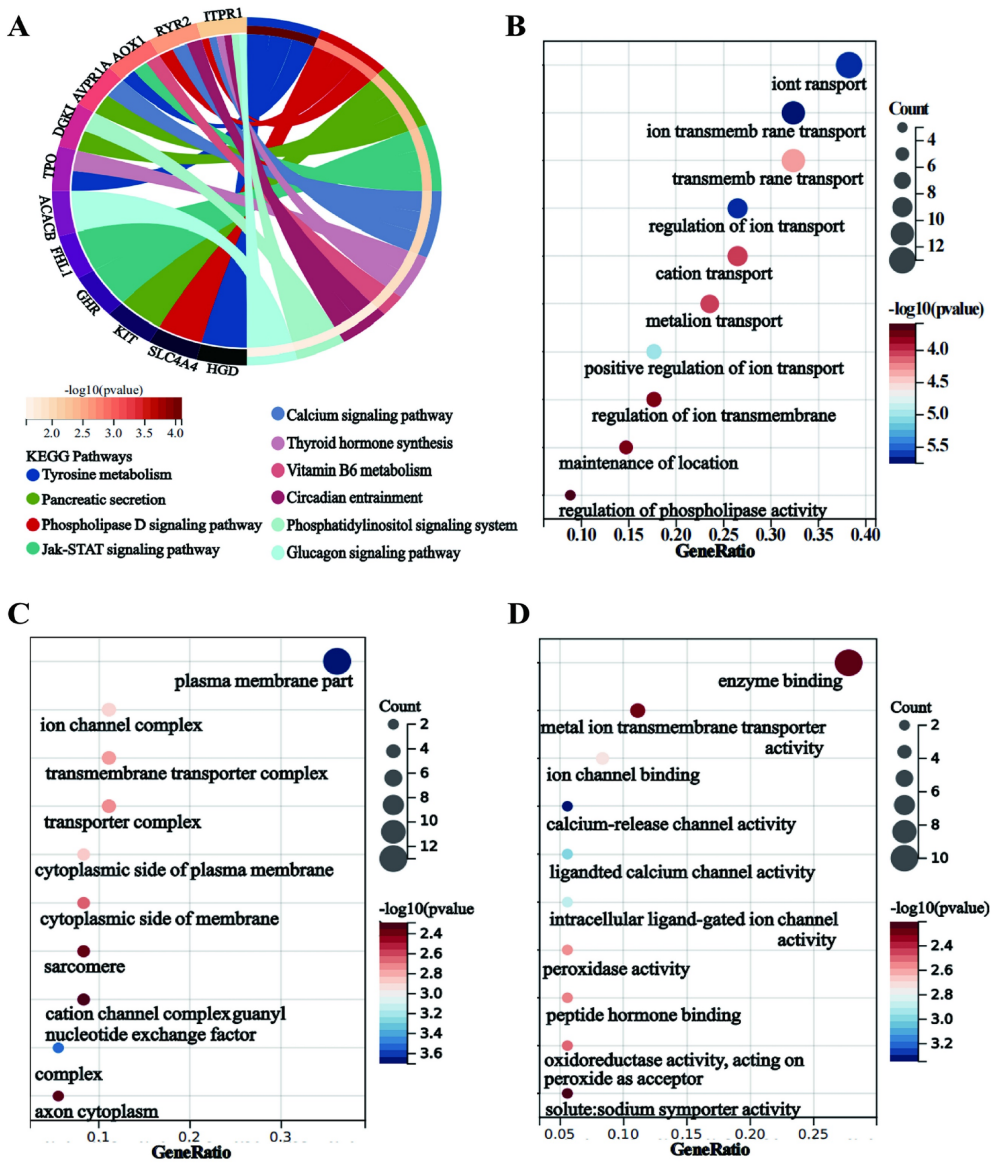
** KEGG pathway and GO enrichment analyses.** (A) KEGG pathway enrichment analyses of *IPCEF1* and its co-expressed genes. (B-D) GO enrichment analyses of *IPCEF1* and its co-expressed genes. (B) Biological Process; (C) Cellular Component; (D) Molecular Function).

To investigate the mechanisms underlying the poor prognosis associated with reduced *IPCEF1* expression, we identified genes correlated with *IPCEF1* in PTC patients and selected the top 50 based on their correlation coefficients. Subsequently, KEGG and GO enrichment analyses were conducted on these 50 genes co-expressed with *IPCEF1* (Figure 8A-I). KEGG pathway enrichment analyses revealed 10 signaling pathways associated with PTC (p < 0.05) (Figure 9A), such as “Tyrosine metabolism,” “Pancreatic secretion,” “Phospholipase D signaling pathway,” and the “Jak-STAT signaling pathway.” Within the biological processes (BP) category of GO enrichment, terms associated with substance transport and metabolism were prevalent, including ion transport, ion transmembrane transport, and regulation of ion transmembrane transport. The molecular function (MF) category indicated that genes associated with *IPCEF1* were primarily involved in enzyme binding, metal ion transmembrane transporter activity, and ion channel binding. In the cellular components (CC) category, *IPCEF1*-associated genes were predominantly found in the plasma membrane, ion channel complexes, transmembrane transporter complexes, and the cytoplasmic side of the plasma membrane. These findings imply that genes associated with *IPCEF1* may contribute to tumor progression by modulating these functions and pathways in PTC.

### *IPCEF1* might influence PTC patient prognosis and survival through immune infiltration

**Figure 10:**
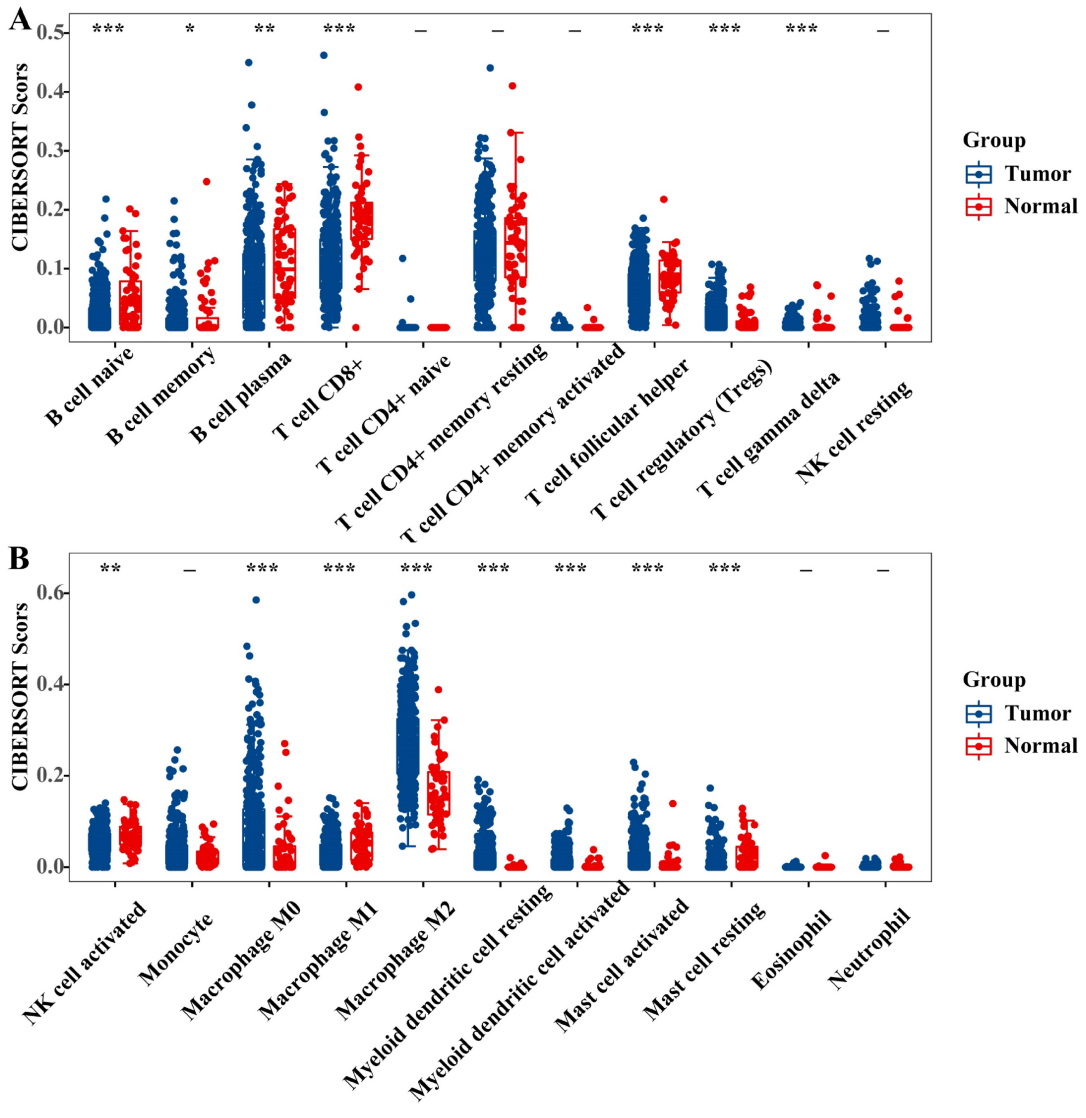
** Immune cell score of PTC tumor and adjacent normal tissues (TCGA) (A-B).** The x-axis represents immune cell types, and the y-axis represents the expression distribution of immune score in different groups (*P < 0.05; **P < 0.01; and ***P < 0.001).

It is recognized that the infiltration of immune cells plays a pivotal role in tumor progression and impacts the prognosis of cancer patients. To delve deeper into the prognostic mechanisms for PTC patients, we employed the 'immuneeconv' function within R software and utilized CIBERSORT to calculate immune cell scores. Our analysis unveiled substantial variances in the infiltration profiles of diverse immune cells when comparing PTC tumors to normal tissue. Specifically, tumor tissues exhibited elevated levels of regulatory T cells (Tregs), M0 and M2 macrophages, both resting and activated myeloid dendritic cells, and activated mast cells. Conversely, normal tissues were characterized by a higher presence of naive B cells, memory B cells, plasma B cells, CD8+ T cells, follicular helper T cells, gamma delta T cells, activated NK cells, M1 macrophages, and resting mast cells (Figure 10A-B).”

**Figure 11:**
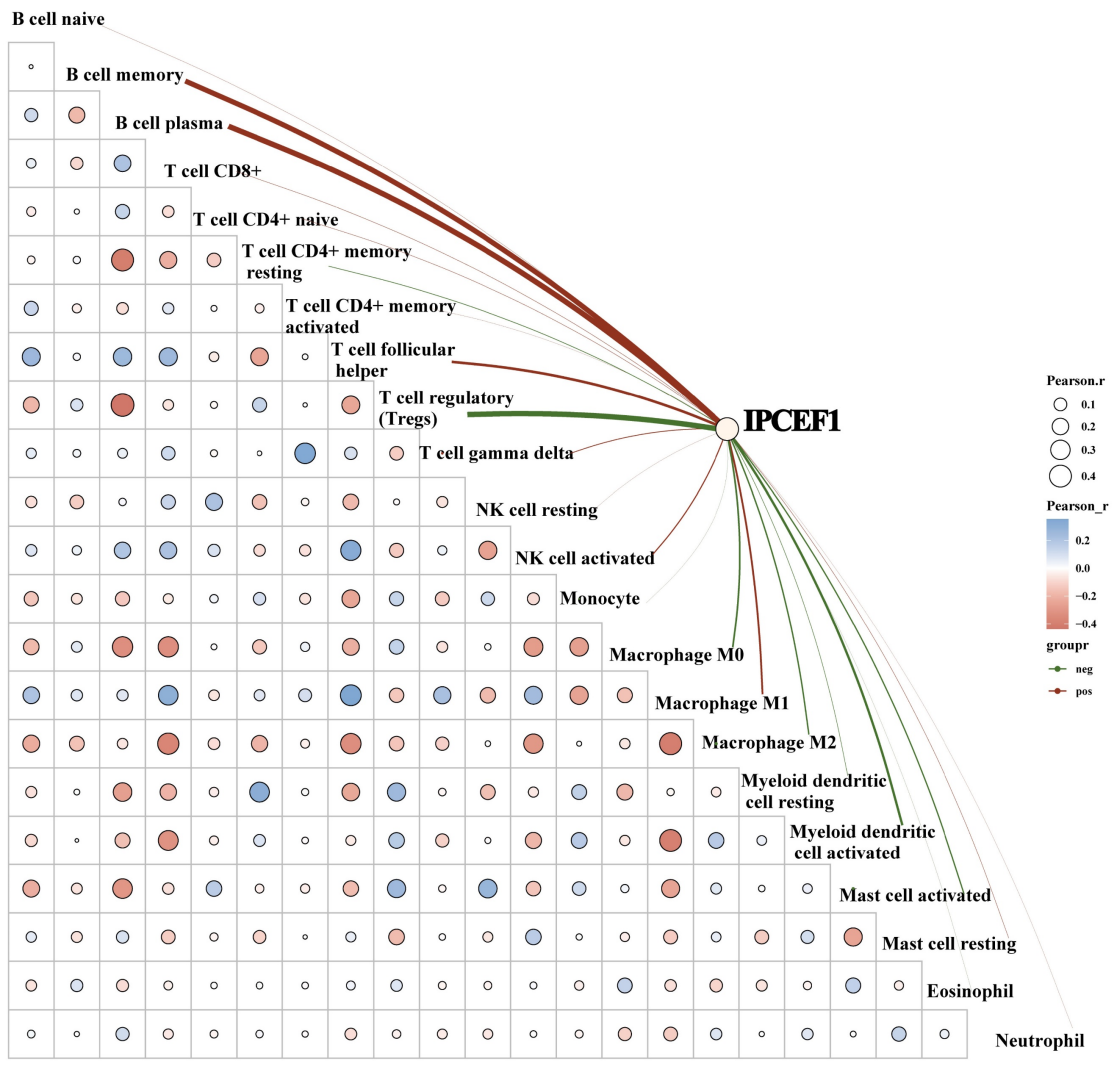
** The correlation between IPCEF1 and immune cells in PTC (TCGA).** The heat map in the schematic represents the correlation analysis of the immune score itself, red represents positive correlation, blue represents negative correlation, the more red or blue color means the greater correlation, also the larger circle means the stronger correlation; the red line in the schematic represents the positive correlation between IPCEF1 expression and the immune score, green means the negative correlation. (*P < 0.05).

To further elucidate whether *IPCEF1* was involved in immune cell infiltration in PTC patients, we conducted a correlation analysis between the expression level of *IPCEF1* and immune cells. Our results indicated a positive correlation between *IPCEF1* expression level and the immune scores for naive B cells, plasma B cells, follicular helper T cells, activated NK cells, and macrophages M1. Conversely, a negative correlation was observed between *IPCEF1* expression level and the immune scores for regulatory T cells (Tregs), M0 and M2 macrophages, activated myeloid dendritic cells, and activated mast cells (Figure 11). These correlations provide additional insight into how *IPCEF1* influence prognosis and survival of PTC patients through mechanisms of immune infiltration.

## Discussion

Previous studies have indicated that low expression of *IPCEF1* might contribute to the progression of several types of cancer and other diseases 8, 14, 15. Yet, its specific function in thyroid cancer, particularly in PTC, is not well-defined. Recent evidence has implicated the abnormal expression of various genes in both the development and progression of PTC. Identifying key molecular players in this cancer type is essential for advancing our understanding of its pathogenesis and developing more effective therapies. This study postulates that overexpression of *IPCEF1* might inhibit PTC progression. We aim to validate this hypothesis by examining the association between *IPCEF1* expression levels and PTC clinical parameters, exploring the effect of *IPCEF1* expression levels on the function of PTC cells, while also investigating the potential mechanisms driving this relationship.

*IPCEF1* is known to interact with cytohesin family members, which are involved in cell adhesion and migration, processes crucial for cancer metastasis 16, 17. Our analyses of TCGA/GEO databases, supplemented by immunohistochemical findings, consistently demonstrated lower *IPCEF1* expression in PTC tumors compared to normal tissue. Echoing this, Ren, S's utilization of bioinformatics and Internet of Things technologies in cancer patient disease analysis revealed a correlation between higher *IPCEF1* expressions with extended survival in thyroid cancer patients 7. Our comprehensive data analysis of PTC patients from the TCGA and GEO databases further revealed that lower *IPCEF1* expression correlates not only with poorer T/N grade and advanced stage but also with reduced disease-free survival (DFS) and progression-free survival (PFS). These findings suggest that *IPCEF1* may be a critical gene influencing PTC progression, corroborating the results obtained by Ren, S. Our cellular level experimental results showed *IPCEF1* reduced the proliferation and migration ability of PTC cells, which is consistent with our clinical observations. Our research showed that overexpression of *IPCEF1* could inhibit.

EMT of PTC cells, which was closely related to the progression of malignant tumor invasion and metastasis. Besides, we also found that high expression level of *IPCEF1* lead to cell cycle arrest in the S or G2 phase, which ultimately reduced cell growth and proliferation.

In our investigation into the action mechanism of *IPCEF1* in PTC, KEGG analysis results showed that the co-expressed genes of *IPCEF1* were enriched in the JAK/STAT signaling pathway. This means that *IPCEF1* may play a role in PTC by regulating the JAK/STAT signaling pathway. The JAK/STAT signaling pathway was discovered 30 years ago 18, 19. As a fulcrum of many important cellular processes, the JAK/STAT pathway constitutes a rapid membrane to nucleus signaling module and induces the expression of key mediators in various cancers and inflammations. More and more evidence suggest that dysregulation of the JAK/STAT pathway is associated with various cancers and autoimmune diseases 20.

The JAK/STAT signaling pathway was evolutionarily conserved, which included ligand receptor complex, JAKs (JAK1/2/3 and Tyk2), and STATs (STAT1/2/3/4/5a/5b/6). Cytokines serve as the primary activators of this pathway, binding to membrane receptors to initiate JAK activation, which in turn mediates the phosphorylation of STATs. These phosphorylated STATs translocate into the nucleus to regulate the transcription of target genes 20. When different stimulus signals were present, the JAK/STAT pathway could mediate different effects and exerted dual anti-tumor and pro-tumor effects based on multiple signals in tumor microenvironment (TME) 21-24. TME is the area around tumor cells that has not undergone transformation, mainly composed of cancer-related fibroblasts and immune cells, provides the contextual landscape for these effects 25. Numerous studies have demonstrated that JAK/STAT signaling pathway could influence tumor prognosis and the efficacy of treatments by modulating the tumor immune microenvironment 26, 27.

To delve deeper into the role of *IPCEF1* in PTC, we assessed the infiltration of immune cells in the tumor microenvironment and analyzed the correlation between *IPCEF1* levels and different immune cell populations. Our findings indicated a negative correlation between *IPCEF1* expression and M2 macrophage infiltration, which was higher in tumor tissues compared to normal tissues. Conversely, *IPCEF1* expression was positively correlated with M1 macrophage infiltration, typically more abundant in normal tissues than in tumor tissues. Macrophages, a predominant component of infiltrating immune cells, are classified into M1 (classically activated) and M2 (alternatively activated) subtypes 28. M1 macrophages are known for their tumor-fighting capabilities, whereas M2 macrophages generally promote tumor development and growth 29, 30. Our data suggest that *IPCEF1* may facilitate the polarization towards M1 macrophages, thereby potentially hampering tumor progression in PTC. This hypothesis is in line with findings from Lu, Y, who demonstrated that Monotropein could inhibit colitis-associated cancer through the VDR/JAK1/STAT1pathway, influencing macrophage polarization^27^. Similarly, Xu, Z's research on osteopontin revealed its role in promoting M1 macrophage polarization via activating the JAK1/STAT1/HMGB1 signaling pathway in Nonalcoholic Fatty Liver Disease 31. These studies complement our results, further suggesting that *IPCEF1* might regulate M1/M2 polarization through the JAK/STAT signaling pathway, thereby impacting the prognosis of PTC.

In addition to macrophages, T cells, which are classified into T cell regulatory (Treg), cytotoxic T cells, and helper T cells based on their cellular functions, are also crucial in tumor related immune cells. Our results showed that *IPCEF1* was negatively correlated with Tregs, which infiltrated more in tumor tissues than that in normal tissues. Tregs were dysregulated in the tumor microenvironment and are key participants in tumor immune escape 32. Tregs were associated with poor prognosis in solid tumor patients and were believed to inhibit anti-tumor immune responses by directly interfering with cytotoxic T cell function, thereby promoting tumor progression and metastasis 33, 34. This pattern suggests that the prognostic significance of *IPCEF1* in PTC may not only be attributed to its influence on macrophage polarization but also to its potential role in modulating Treg populations. A lower expression of *IPCEF1* may contribute to a more favorable prognosis by reducing Treg-mediated suppression of anti-tumor immunity.

Globally, thyroid cancer is among the top ten commonest cancer in females. In both adult and pediatric populations, there are variations of prevalence of thyroid cancer and rising incidence rates of thyroid cancer worldwide 35. Despite the generally favorable prognosis of PTC, it can still exhibit aggressive behavior and lead to patient mortality 2, 3. The various adverse reactions of targeted drugs currently make the development of new targets and agents urgent. In this context, the association of high *IPCEF1* expression with improved prognosis in PTC patients emerges as a promising avenue for developing novel targeted treatment strategies. Nonetheless, the design of appropriate delivery carriers and the evaluation of their therapeutic efficacy warrant further investigation.

Collectively, our results suggested that *IPCEF1* was lower expression of PTC tumor tissues than that in normal tissues and its lower expression might led to worse T/N stage and DFS/ PFS, which perhaps related to its regulation of the JAK/STAT signaling pathway and immune microenvironment (macrophage and Tregs). Of course, these results still require final basic experiments to confirm, which is also the focus of our subsequent research. But these results at least gave us a way of thinking that using exosomes or biological nanomaterials to target *IPCEF1* delivery to the lesion site may be a new approach to inhibiting PTC progression, which still requires us to continue exploring.

## Conclusion

Our findings suggested that *IPCEF1* is a cancer suppressor gene in the progression of PTC, influencing patient survival and prognosis through modulation of immune infiltration and signaling pathways. These insights pave the way for novel therapeutic strategies, such as targeted delivery of *IPCEF1* using exosomes or biomimetic nanomaterials. Although further validation is required, this research offers a new perspective for future PTC treatment modalities.

## Figures and Tables

**Table 1:** The association between *IPCEF1* expression levels and clinicopathological characteristics of PTC (TCGA).

	*IPCEF1* High expression (N=199)	*IPCEF1* Low expression (N=198)	Total	Chi-square	P
			(N=397)		
**Age**				3.099	0.078
>45 year	95 (12.59%)	112 (17.38%)	119 (29.97%)		
≤45 year	104 (37.53%)	86 (32.49%)	278 (70.03%)		
**Gender**				1.608	0.205
Male	49 (12.34%)	60 (15.11%)	109 (27.46%)		
Female	150 (37.78%)	138 (34.76%)	288 (72.54%)		
**T**				14.36	0.0025
I	74 (18.23%)	59 (14.53%)	111 (32.76%)		
II	65 (16.01%)	57 (14.04%)	123 (30.05%)		
III	54 (13.30%)	84 (20.69%)	138 (34.09%)		
IV	2 (0.49%)	11 (2.71%)	13 (3.2%)		
I~II	139 (35.01%)	97 (24.43%)	236 (59.44%)	17.91	<0.0001
III~IV	60 (15.11%)	101 (25.44%)	161 (40.56%)		
**N**				16.751	<0.0001
N0	110 (27.71%)	68 (17.13%)	178 (44.84%)		
N1	89(22.42%)	130 (32.75%)	219 (55.16%)		
**M**				--	0.3718
M0	198 (49.87%)	195 (49.12%)	393 (98.99%)		
M1	1 (0.25%)	3 (0.76%)	4 (1.01%)		
**Stage (TNM)**				17.26	0.0006
I	127 (32.23%)	96 (24.37%)	223 (55.6%)		
II	17 (4.32%)	12 (3.05%)	29 (7.37%)		
III	41 (10.41%)	52 (13.2%)	93 (23.61%)		
IV	13 (3.3%)	36 (9.14%)	49 (12.44%)		
I~II	144 (36.27%)	110 (27.71%)	254 (63.98%)	12.161	0.0005
III~IV	55 (13.85%)	88 (22.17%)	143 (36.02%)		

## Abbreviations

PTC: Papillary Thyroid Cancer
IPCEF1: Interaction Protein for Cytohesin Exchange Factors 1
GEO: Gene Expression Omnibus
DEG: Differentially Expressed Genes
TCGA: The Cancer Genome Atlas
THCA: Thyroid Cancer
OS: Overall Survival
DFS: Disease-Free Survival
PFS: Progression-Free Survival
IHC: Immunohistochemical analysis
PBS: Phosphate Buffered Saline
MMP9: Matrix Metallopeptidase 9
SNAL1: Snail Family Transcriptional Repressor 1
GAPDH: Glyceraldehyde-3-Phosphate Dehydrogenase
KEGG: Kyoto Encyclopedia of Genes and Genomes
GO: Gene Ontology
